# The Role of Lisinopril and Bisoprolol to Prevent Anthracycline Induced Cardiotoxicity in Locally Advanced Breast Cancer Patients

**DOI:** 10.31557/APJCP.2021.22.9.2847

**Published:** 2021-09

**Authors:** Asdi Wihandono, Yohana Azhar, Maman Abdurahman, Sjarief Hidayat

**Affiliations:** 1 *Medical Faculty, Universitas Airlangga, Indonesia. *; 2 *Department of Surgery, Faculty of Medicine, Universitas Padjadjaran, Bandung, Indonesia. *; 3 *Department of Cardiology and Vascular Medicine, RSUP Dr Hasan Sadikin Hospital Bandung, Indonesia. *

**Keywords:** Anthracycline, Cardiotoxicity, Lisinopril, Bisoprolol

## Abstract

**Background:**

Anthracyclines are a class of chemotherapeutic agents that are used to treat many different cancers, including breast cancer. Although anthracyclines remain an effective and commonly used therapy, their use is limited by cardiotoxicity. Heart failure and left ventricular (LV) dysfunction are the short and long-term complications of anthracyline exposure occurring in 5% to 23% of patients. Recent prospective studies have investigated the prophylactic role of ACE inhibitors and beta-blockers as cardioprotective agents. This study aimed to evaluate whether the addition of lisinopril and bisoprolol could prevent anthracycline induced cardiotoxicity.

**Methods:**

In this randomized, controlled trial, 74 subjects with locally advanced breast cancer were randomly assigned to a group receiving lisinopril and bisoprolol (n=37) or to a control group (n=37). Lisinopril and bisoprolol was started simultaneously 24 h before the first cycle of chemotherapy. The initial dose was 2.5 mg each, once daily, and was increased gradually under close supervision to 10 mg if SBP persistently remained >90 mmHg and HR >60 bpm. Echocardiographic studies were performed before and after the 6th cycle of neoadjuvant anthracycline-based chemotherapy (FAC). The primary endpoint was the change from baseline LVEF.

**Results:**

There was no difference in baseline LVEF between intervention and control group (65.77 ± 4.56 % v 65.64 ± 455 %, p = 0.92). There was also no difference in total anthracycline doses between 2 groups (579.48 ± 65.10 mg vs 557.50 ± 47.76 mg, p = 0.18). However, after 6 cycles of FAC, the rate of decline in LVEF was greater in control group (-5.52 ± 8,90 %) than in the intervention group (-0.27 ± 5.73 %) with p = 0.017. No severe adverse effects occurred in the intervention group related to the treatment with lisinopril and bisoprolol.

**Conclusion:**

Combined treatment with lisinopril and bisoprolol may prevent anthracycline-induced cardiotoxicity in patients with locally advanced breast cancer treated with anthracycline-based chemotherapy. The clinical relevance of this study should be confirmed in larger studies with longer follow up time.

## Introduction

The American Cancer Society (2016) estimated, that there were 3.5 million women in the United States diagnosed with invasive breast carcinoma. Of these, about 90% will survive more than 5 years after diagnosis and become cancer survivors (Siegel et al., 2013). Cardiovascular disease (CVD) is the leading cause of death in breast cancer survivors (Schairer et al., 2004), and patients with cardiovascular risk factors have higher risk of developing it (Patnaik et al., 2011; Weaver et al., 2013). When patients begin to enter the period of long-term survivorship (generally more than 5 years after diagnosis), mortality due to CVD equals, or even higher than the primary disease (Swiger, 2016). This is due to cardiotoxicity, which is related to the direct effect of cancer therapy, affecting the structure and function of cardiac tissue, or due to the acceleration of CVD development, especially in patients with risk factors for CVD (Armstrong et al., 2013).

Chemotherapy has a wide spectrum of side effects on the cardiovascular system including cardiac dysfunction, heart failure, cardiac ischemia, arrhythmias, pericarditis, prolonged QTc interval, hypertension and thromboembolism (Yeh and Bickford, 2009; Thakur and Whitteles, 2014). Certain types of cancer drugs that have been reported to cause cardiac dysfunction include anthracyclines, trastuzumab, and several types of tyrosine kinase inhibitors (Swain et al., 2003; Colombo et al., 2013).

The prevention and management of cardiotoxicity caused by anti-cancer drugs has been the subject of research in recent years (Hensley et al., 2009; Abdel-Rahman and Fouad, 2014). Several methods have been studied to reduce the risk of cardiac dysfunction, including limiting the cumulative dose of anthracyclines, regulating the rate of anthracycline administration, changing anthracycline formulations, and the utilization of cardioprotective drugs (Geisberg and Sawyer, 2010). This study will examine the benefits of adding ACEi (lisinopril) and beta-blocker (bisoprolol), to reduce the cardiotoxicity of breast cancer patients receiving anthracycline chemotherapy.

## Materials and Methods

Trial. This was an open-label prospective randomized controlled trial performed at the dr. Hasan Sadikin General Hospital in Bandung, Indonesia. All patients were informed orally and in writing, and all gave their written consent before inclusion. The protocol was approved by the ethic committee of our institution that recommended an open-label design of the study considering the severity of the treated disease, the high incidence of infectious complications, and the potential hipotensive effect of the intervention. The study was conducted according to the Helsinki declaration.

Population of the study. Inclusion criteria were adult patients from 18 to 70 years old, in sinus rhythm and normal echocardiographic LV ejection fraction (LVEF ≥50%), recently diagnosed with locally advanced breast cancer who were submitted to receive anthracycline-based neoadjuvant chemotherapy. Exclusion criteria were patients with history of cardiovascular disease (congestive heart failure, myocardial infarction, coronary artery disease, heart valve disease or cardiomyopathy); ongoing or expected to be treated with lisinopril and or bisoprolol for other diseases; allergic to lisinopril or bisoprolol; systolic blood pressure (SBP) lower than 90 mmHg; heart rate lower than 60 beats per minute, pregnancy; and unable or unwilling to give informed consent.

Randomization. Participants were randomly assigned in a 1:1 ratio to receive (intervention arm) or not to receive (control arm) lisinopril and bisoprolol. Randomization was aided by a randomization table generated by a computer program in blocks of random size.

Study treatment. Lisinopril and bisoprolol was started simultaneously at least 24 hours before the first cycle of chemotherapy on the treatment arm. The initial dose of lisinopril was 2,5 mg once daily, and was increased gradually every 7 days to a maximum dose of 10 mg, if SBP persistently remain >90 mmHg and serum creatinine levels <2,5 mg/dl. In the case of hypotension, the dose was reduced to the closest level or stopped, and the lowest dose resumed when SBP persistently remained >90 mm Hg. The initial dose of bisoprolol was 1,25 mg once daily and increased gradually every 7 days to a maximum dose of 10 mg in the absence of clinical signs of congestive heart failure, sinus bradicardia <60 beats per minute or any degree of AV block. In the case of hypotension or bradicardia, dose was also reduced to the closest level. All patients received in-hospital chemotherapy according to the protocols of our institution.

Echocardiography. Echocardiographic studies were performed using Vivid 7 Ultrasound Machine, General Electric Medical System. Images were digitally stored for later offline analysis with EchoPac, General Electric. LVEF was calculated using the Simpson’s method. LVEF measurement were evaluated before the first cycle and after the 6^th^ cycle of chemotherapy.

Sample size and statistical analysis. To detect an intergroup difference of 5 points in LVEF change from baseline to post chemotherapy with a statistical power of 90%, a type I error risk of 5%, and with an estimated standard deviation (SD) of 6,5%, 29 patients was estimated to be needed on the basis of a 2-sided, 2-sample t-test. Assuming a 30% rate of incomplete measurements, a total of 75 patients was needed to be enrolled in the study.

Statistical analysis. All statistical analysis were done using the SPSS software v.25. Descriptive statistics were presented as number and percentage for categorical data, and mean, SD and range for numerical data. Normality of the numerical data was evaluated with Shapiro-Wilk test. Analytical statistics were performed using a) Bivariate analysis with unpaired t-test, since the numerical data is normally distributed; and b) Multivariate analysis with linear regression (backward method) to assess whether an inhomogenous independent variable could affect a dependent variable.

## Results

**Figure 1:**
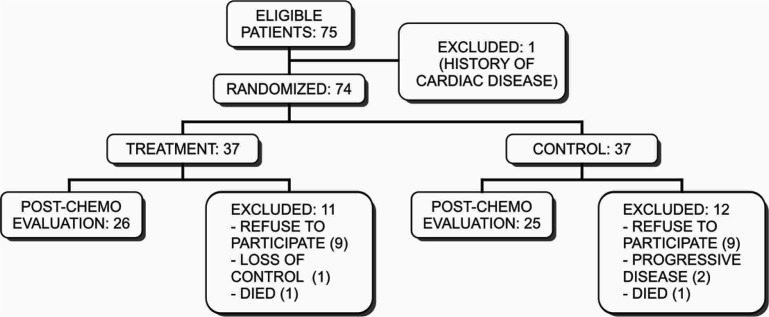
Flow Diagram of the Study

The study was conducted on 51 women with locally advanced breast cancer (LABC) who met the inclusion criteria and followed until the study completed (Figure 1). Characteristics of subjects according to breast cancer stage, according to the 2017 AJCC TNM criteria, 24 (92.3%) subjects in the treatment group were stage IIIB LABC patients, while 2 subjects (7.7%) were in stage IIIC. In the control group, all subjects (25; 100%) were in stage IIIB (Table 1).

Comparable characteristics were obtained in two arms in terms of body mass index (BMI), body surface area (BSA), systolic blood pressure (SBP), diastolic (DBP), heart rate (HR), total dose of doxorubicin given, as well as LVEF value before chemotherapy. For the age variable, the mean in the treatment arm was 44.5 ± 7.78 years, while in the control arm it was 50.8 ± 7.39 years (Table 2).

In the treatment arm, each patient received lisinopril and bisoprolol tablets that were taken once a day. The dosage for each patient is adjusted according to initial blood pressure and heart rate before the first chemotherapy cycle is given. The average dose of lisinopril was 7.92 mg/day while the bisoprolol dose was 6.47 ± 1.07 mg/day. All patients in the treatment arm were given the combination of lisinopril and bisoprolol until the end of the study.

**Figure 2:**
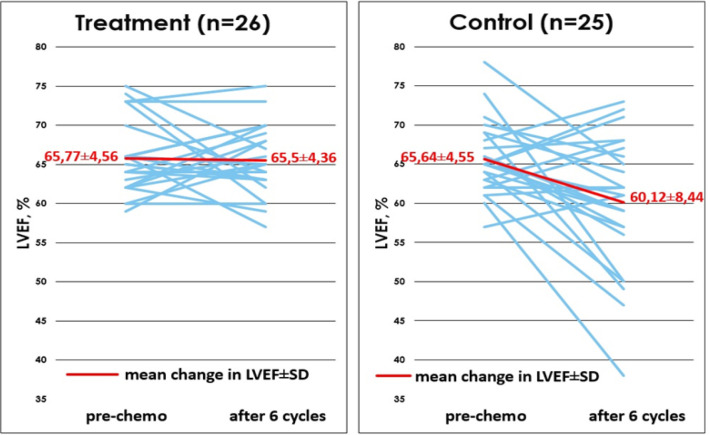
Comparison of Changes in LVEF between Treatment and Control Arms

The mean baseline LVEF value obtained from echocardiography was comparable in two arms, with the results of the treatment arm being 65.77 ± 4.56, and in the control arm 65.64 ± 4.55 (p=0.92). The mean value of LVEF after 6th chemotherapy cycle for the treatment arm was 65.5 ± 4.36, while in the control arm was 60.12 ± 8.44. Mean change in LVEF as measured after the 6th chemotherapy cycle was -0.27 ± 5.73 in the treatment arm, and -5.52 ± 8.90 in the control arm. There was a significant difference (p=0.017) in LVEF change between control arm and the treatment arm. The comparison of changes in LVEF in each arm can be seen in Figure 2.

**Figure 3:**
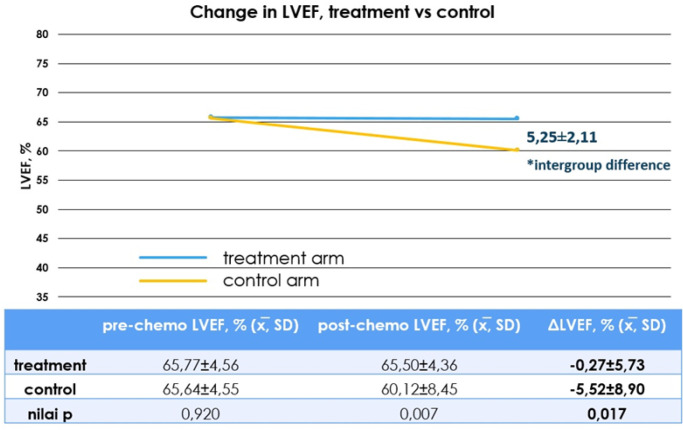
Mean Change in LVEF and Statistical Analysis

To analyze the difference in the LVEF changes between the two arms, an unpaired t-test was used, because the data for the two groups were normally distributed. From the unpaired t-test the result was p=0.017. Because p<0.05, there is a significant difference in LVEF change between control and treatment arms (Figure 3).

Based on the characteristics of the subject, there was a significant difference between the mean age of the subject in the treatment and control arm (p=0.003), with the mean age in the treatment arm is 44.5 ± 7.78 years, and 50.8 ± 7.39 years in control arm. To test whether this significant age difference can affect changes in the value of LVEF, a linear regression test was performed using the backward method. The linear test results showed changes in R2 and coefficient B respectively 0.005 (0.5%) and 0.077 (7.7%). Because those changes are below 10%, it can be concluded that the age factor is not a confounding factor for changes in the LVEF value (Table 3).

**Table 1:** Characteristics of Research Subjects Based on stage, Histopathological Type and Immunohistochemical Subtypes

	Intervention Group (n=26)	Control (n=25)
Stage		
IIIB, n (%)	24 (92,3)	25 (100)
IIIC, n (%)	2 (7,7)	0
Histopathology		
ICNST, n (%)	4 (15,4)	1 (4,0)
ICNST I, n (%)	1 (3,8)	1 (4,0)
ICNST II, n (%)	10 (38,5)	11 (44,0)
ICNST III, n (%)	9 (34,6)	8 (32,0)
ILCM, n (%)	0	1 (4,0)
ILCM II, n (%)	2 (7,7)	1 (4,0)
Medullary, n (%)	0	1 (4,0)
Metaplastic, n (%)	0	1 (4,0)
IHC subtype		
Luminal A, n (%)	1 (3,8)	0
Luminal B, n (%)	14 (53,8)	13 (52,0)
HER2(+), n (%)	8 (30,8)	6 (24,0)
HER2(-), n (%)	6 (23,1)	7 (28,0)
HER2 overexpression	5 (19,2)	7 (28,0)
Basal-like, n (%)	6 (23,1)	5 (20,0)

**Table 2:** Baseline Clinical Differences between Groups

	Intervention Group (n=26)	Control Group (n=25)	p
Age, years (x̅, SD)	44,5±7,7	50,8±7,39	0,003*
min-max	32-61	37-64	
BMI (x̅, SD)	25,61±4,75	24,22±3,84	0.256
BSA, m² (x̅, SD)	1,62±0,18	1,56±0,14	0.252
SBP, mmHg (x̅, SD)	129,31±15,56	125,80±14,46	0.409
DBP, mmHg (x̅, SD)	84,31±11,65	83,24±10,92	0.737
HR, x/min (x̅, SD)	91,27±13,96	89,44±12,25	0.622
Total dose of Doxorubicin, mg (x̅, SD)	579,48±65,10	557,50±47,76	0.177
LVEF pre-chemo, % (x̅, SD)	65,77±4,56	65,44±4,55	0.92

**Table 3:** Linear Regression of the Age Variable

Variable	Age analyzed		Age not analyzed		ΔR²	Change in coefficient B
	R²	Coefficient B	R²	Coefficient B		
Treatment/control	0.119	5.692	0.114	5.251	0.005	0.077
Age		0.07		-		-

## Discussion

Anthracyclines are effective chemotherapy agents with anti-tumor mechanisms in 4 ways. Anthracyclines interfere with DNA and RNA synthesis by forming complexes between base pairs (intercalating), inhibiting topoisomerase II and causing DNA damage and inhibiting ligase repair (Geisberg and Sawyer, 2010), causing the release of histone from chromatin, thereby disrupting the function of DNA repair (Pang et al., 2013), and making iron-mediated free radicals (anthracycline-Fe^2+^ or anthracycline-Fe^3+^ complexes) which will damage DNA strands (Zhang et al., 2012; Abdel-Rahman and Alorabi, 2015).

Anthracycline-induced cardiotoxicity (AIC) has been studied in recent decades. Anthracyclines trigger cellular injury to heart muscle cells and endothelial cells through excess free radicals produced by the quinone group (Horenstein et al., 2000). Topoisomerase 2 (Top2) plays a role not only in the anticancer mechanism of anthracyclines, but also in AIC. DNA topoisomerase is an important enzyme required for DNA transcription, replication and recombination. The two Top2 isoenzymes expressed in humans are Top2α and Top2β. Top2α is mostly expressed in cells with high proliferation rates such as cancer cells, whereas Top2β is expressed on quiescent cells such as myocardium. In one study, mice that did not express Top2β were not affected by anthracycline-induced cardiotoxocity (Lyu et al., 2007).

The focus of cardiotoxicity has recently changed from treatment to prevention (Hensley et al., 2009; Abdel-Rahman and Fouad, 2014). Several strategies have been used, including modification of the schedule for anthracycline administration (long-term infusion is safer than rapid administration), liposomal packaging of anthracyclines, and administration of cardioprotector drugs such as dexrazoxane, ACE inhibitors, angiotensin receptor blockers (ARBs), beta-blockers, and several others (Geisberg and Sawyer, 2010). Research by Bosch et al. (2013) stated that the combination of enalapril and carvedilol was effective in preventing chemotherapy-induced left ventricular systolic dysfunction (LVSD) in patients with haematological malignancies.

Angiotensin-converting enzyme (ACE) inhibitor (ACEi) has been reported to slow the process of LV dysfunction in several clinical conditions, including anthracycline-induced cardiomyopathy (Yusuf et al., 1992; Jensen et al., 1996; López-Sendón et al., 2004; Hunt, 2005). Other studies has reported the role of renin-angiotensin system in anthracycline-induced cardiomyopathy and that administration of ACEi can protect heart muscle cells from chemotherapy-induced cardiotoxicity (Maeda et al., 1997; al-Shabanah et al., 1998; Tokudome et al., 2000; Abd El-Aziz et al., 2001; Sacco et al., 2001; Okumura et al., 2002; Vaynblat et al., 2002).

Boucek et al. (2003) evaluated the effect of lisinopril on delayed-onset anthracycline-induced cardiotoxicity in rabbits. From this study, it was found that lisinopril can prevent increased expression of ventricular atrial natriuretic peptide (ANP) and prevent anthracycline-induced myocyte loss. Several studies of ACEi as a cardioprotector are still ongoing, including the comparison of lisinopril and carvedilol extended release (Coreg CR®) in breast cancer patients who received trastuzumab (Boucek et al., 2003). Lisinopril has the same tolerance profile as other ACEi, has a stable effect within 24 hours with a single administration, so it can increase patient compliance, and requires lower cost than other ACEi (Leonetti and Cuspidi, 1995; Goa et al., 1996).

Likewise, beta-adrenergic receptor antagonists (beta-blockers) have been reported in several studies to reduce the cardiotoxic effects of anthracyclines. Kaya et al., (2013) reported a decrease in LVEF in the control group, whereas in the treatment group given nebivolol (β1-selective receptor antagonist), the decrease was lower. Kalay et al., (2006) reported that carvedilol reduced ejection fraction in patients receiving anthracycline chemotherapy. Administration of beta-blockers in patients with cardiac dysfunction improved left ventricular function. Beta-blockers not only block the cardiac remodeling process, they can also repair it. The Cardiac Insufficiency Bisoprolol Study (CIBIS)-I showed the use of bisoprolol can reduce hospitalization rates, pulmonary edema incidence, ventricular tachycardia (VT) and ventricular fibrillation (VF) incidence in heart failure patients (Circulation, 1994). CIBIS-II study with a larger sample has shown that bisoprolol use can reduce mortality, sudden cardiac death and hospitalization rates in patients with heart failure (Lancet, 1999).

The MANTICORE (Multidisciplinary Approach to Novel Therapies in Cardiology Oncology Research) study was the first to examine whether administration of ACEi (perindopril) or beta-blockers (bisoprolol) can reduce the cardiotoxic effects of trastuzumab in HER-2 positive early breast cancer patients. As a result of this study, there was a significant reduction in LVEF in the treatment arm (a marker of cardiomyopathy), which did not occur in the control arm (Pituskin et al., 2017).

The combination of ACEi and beta-blockers has been reported by some researchers to reduce the cardiotoxic effects of anthracyclines. The PRADA (Prevention of Cardiac Dysfunction During Adjuvant Breast Cancer Therapy) study examined whether the use of angiotensin receptor blockers (candesartan) or beta blockers (metoprolol) or a combination of the two could prevent LV dysfunction in early breast cancer patients receiving standard adjuvant therapy (Heck et al., 2012). Results from the study indicated that candesartan could prevent the reduction in LVEF associated with breast cancer adjuvant therapy, but not metoprolol.

The OVERCOME (Prevention of Left Ventricular Dysfunction With Enalapril and Carvedilol in Patients Submitted to Intensive Chemotherapy for the Treatment of Malignant Hemopathies) study showed that prophylactic use of enalapril and carvedilol combination stabilized LVEF compared to the control group after 6 months (Bosch et al., 2013).

In this study, the mean value of LVEF after the 6^th ^chemotherapy cycle for the treatment arm was 65.5±4.36 while in the control arm was 60.12±8.44. Mean change in LVEF as measured after the 6th chemotherapy cycle was -0.27 ± 5.73 in the treatment arm, while in the control arm it was -5.52 ± 8.90. There was a significant difference (p=0.017) in LVEF change between two groups. This is the same as the study conducted by Cardinale et al. (2006) who evaluated the role of ACEi (enalapril 20 mg once daily) for the prevention of high-dose chemotherapy (HDC)-related cardiotoxicity in high-risk cancer patients and found a significant decrease in LVEF and increased volume of end-diastolic and end-systolic was only seen in the control group (p<0.001).

One of the weaknesses of this study is that the age variable has a significant difference in mean between treatment and control arms. Fiechter et al., (2013) have reported LVEF values in normal subjects that did not differ significantly between the 30-49 years (60.8% ± 5.7%) and 50-69 years (65.2% ± 7.0%) age groups with p<0.001. Zamorano et al., (2016) in the ESC Position Paper on cancer treatments and cardiovascular toxicity stated that age is a risk factor for cardiotoxicity due to anthracycline-induced cardiotoxicity, namely age <18 years and >65 years. The two arms in this study similarly have a low risk factor for cardiotoxicity due to chemotherapy based on statement above.

Cardinale (2015) studied 2,625 patients with various types of cancer who received anthracycline-based chemotherapy, and were followed-up for a median of 5.2 years. It was found that 98% of cardiotoxicity (symptomatic reduction in LVEF) occurred in the first 12 months after administration of anthracycline therapy. This study carried out a follow-up until after the 6th chemotherapy, or about 5 months after starting anthracycline chemotherapy. There may still be undocumented events of cardiotoxicity, given the follow-up time of less than 12 months. To obtain accurate data regarding the number and type of cardiotoxicity in patients receiving anthracycline chemotherapy, follow-up of up to at least 12 months after the start of the first chemotherapy cycle is required.

In conclusion, the combination of lisinopril and bisoprolol can prevent cardiotoxicity which is indicated by a decrease in LVEF in patients with locally advanced breast carcinoma receiving anthracycline chemotherapy.

## Author Contribution Statement

Study conception and design: Asdi Wihandono, Yohana Azhar, Maman Abdurahman. Data collection: Asdi Wihandono. Analysis and interpretation of the results: Asdi Wihandono, Yohana Azhar. Draft Manuscript Preparation: Asdi Wihandono, Yohana Azhar, Maman Abdurahman, Sjarief Hidayat. All authors reviewed te results and approved the final version of the manuscript.
